# The Good, Bad, and Ugly of Inferior Vena Cava (IVC) Filters: Anesthetic Management for IVC Filter Retrieval in the Setting of Filter Thrombosis

**DOI:** 10.7759/cureus.22591

**Published:** 2022-02-25

**Authors:** Emily Bergbower, Paul S Park, Seema Deshpande

**Affiliations:** 1 Anesthesiology, University of Maryland Medical Center, Baltimore, USA

**Keywords:** venous thromboembolism (vte), non-operating room locations, ivc filter retrieval, interventional radiology, anesthesiology

## Abstract

We describe a case of a 58-year-old man presenting to the interventional radiology (IR) suite for inferior vena cava (IVC) filter retrieval and potential intravascular iliocaval stent reconstruction in the setting of anticoagulation and uncontrolled hypertension. This patient had recently undergone iliocaval thrombectomy with IVC venoplasty four weeks prior to presentation. Induction of anesthesia and endotracheal intubation occurred without complication. The patient received two large-bore intravenous (IV) catheters and a radial artery catheter for hemodynamic monitoring. Blood was cross-matched and kept in the IR suite, anticipating bleeding from a potential injury to the IVC during filter retrieval. Fortunately, the thrombosed filter was removed without complication. This case illustrates the importance of in-depth anesthetic planning for so-called “benign” surgical procedures and highlights the challenges faced in non-operating room locations for anesthesiologists.

## Introduction

Inferior vena cava (IVC) filters are a mainstay of management for venous thromboembolism (VTE) in patients who suffer recurrences despite anticoagulation or for whom anticoagulation therapy is contraindicated [1]. Significantly, IVC filters are associated with a host of clinical complications including filter thrombosis, infection, IVC thrombosis, IVC penetration, filter embolization and movement, and filter fracture, necessitating removal [1,2]. IVC filters can be removed through a variety of safe endovascular retrieval techniques, but due to severe consequences of the known clinical complications, thorough and advanced preparation for anesthetic management should be employed [3]. Here, we present a case of a 58-year-old man requiring IVC filter retrieval and possible iliocaval stent reconstruction secondary to thrombosis, as well as our careful anesthetic planning in the setting of a non-operating room location.

## Case presentation

A 58-year-old man with a history of hypertension, multiple deep vein thromboses (DVTs), and a prior pulmonary embolus (PE) presented for IVC filter retrieval and potential iliocaval stent reconstruction in the interventional radiology (IR) suite. One month prior, the patient was admitted to the hospital with severe bilateral lower extremity pain, shortness of breath, and nausea. He was subsequently found to have an acute occlusive thrombus of the infrarenal IVC extending to the anterior portion of the IVC filter, as well as bilateral common iliac and common femoral vein DVTs. He underwent iliocaval thrombectomy, IVC venoplasty, and received catheter-based thrombolysis. Subsequently, he was discharged home on anticoagulation with apixaban and instructed to return for IVC filter retrieval and possible iliocaval stent reconstruction four weeks later.

Upon presentation, the patient noted total resolution of the lower extremity pain and shortness of breath present during his previous hospital admission. He confirmed that he was compliant with anticoagulation, having taken apixaban through the morning of surgery, but was completely non-compliant with prescribed anti-hypertensives. In addition to apixaban, his only other home medication was methadone, prescribed for daily maintenance in the setting of former IV drug use. The patient had no known allergies to medication and no history of airway or anesthetic complications. Pre-operatively, his temperature was 35.6°C, heart rate was 74 beats/minute, BP was 144/86 mmHg, and SpO_2_ was 99% on room air. Initial hemoglobin was 10.3 g/dL with a hematocrit of 33.8%. Platelet count, prothrombin time, partial thromboplastin time, and international normalized ratio were all within normal limits. A basic metabolic panel revealed no abnormalities.

Outcome and follow-up

**Figure 1:**
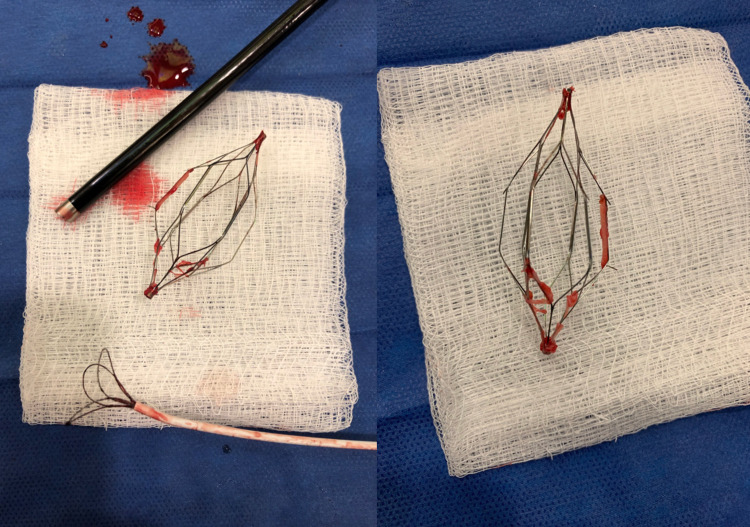
The retrieved inferior vena cava (IVC) filter with a significant clot burden attached to the individual struts.

**Figure 2:**
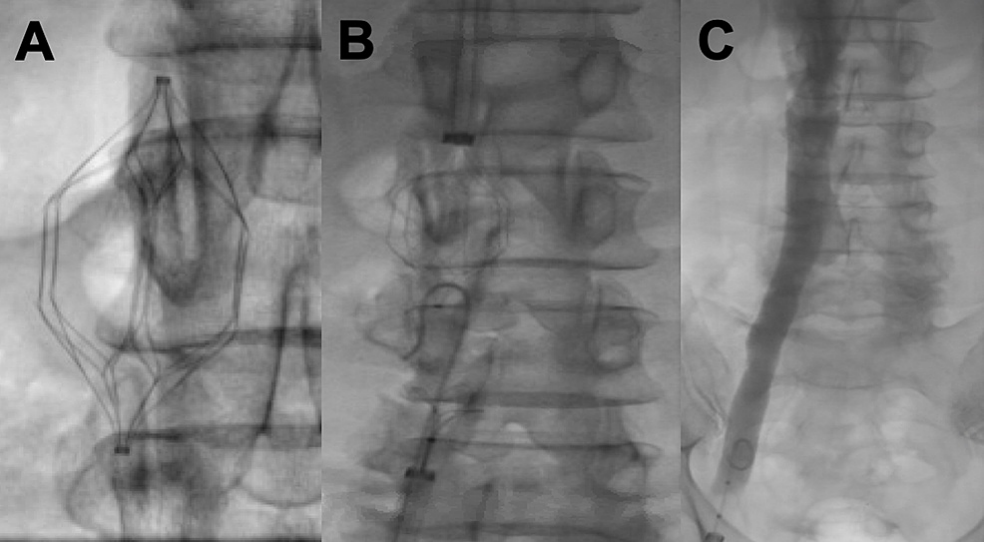
Before, during, and after removal of the TrapEase filter. (A) A view of the filter prior to the procedure starts. (B) Fluoroscopic-guided wire loop and capture of the cranial and caudal ends of the TrapEase filter. (C) Post-procedure venogram showing complete filter removal and a patent right iliac vein and inferior vena cava.

We identified five major concerns for anesthetic planning in this case: the patient's anticoagulated state, uncontrolled hypertension, the possibility for IVC thrombosis, the potential for IVC injury during retrieval, or the need for intravascular iliocaval stent reconstruction, and the non-operating room location. The surgical plan was a fluoroscopic-guided wire loop and capture of the filter, followed by balloon venoplasty of the right common iliac vein and filter retrieval using a laser sheath. Due to concerns that the procedure could become extensive secondary to potential IVC thrombosis or the need for iliocaval stent reconstruction, a general anesthetic technique using an endotracheal tube was employed in place of sedation. Additionally, due to the patient’s anticoagulated state and poorly managed hypertension, blood products were ordered for the IR suite and a radial artery catheter was placed, in addition to two large-bore IVs, for close hemodynamic monitoring and sampling of arterial blood. Available blood products included four units of packed red blood cells and two units of fresh frozen plasma. The induction of anesthesia and endotracheal intubation was smooth and uneventful. The filter was fortunately removed without complication but was noted to have a significant clot burden associated with the struts (Figure 1). The patient remained hemodynamically stable throughout the procedure, requiring only a low-dose phenylephrine infusion for support intermittently. A right iliocaval venogram revealed that no reconstruction was required. Figures 2A-2C display the progression of IVC filter retrieval. Induction of anesthesia, the procedure itself, and emergence was about five hours in length. The patient was safely discharged home after 24 hours of observation.

## Discussion

VTE is a significant source of morbidity and mortality among the patient population in the United States, affecting nearly one million patients each year [4]. While many patients are easily treated with anticoagulation therapy, in those for whom anticoagulation is contraindicated, IVC filters are a mainstay of therapy and have been used successfully to trap venous emboli since 1973 [5,6]. The procedural ease and utility of filter placement have led to a significant rise in the number of these procedures performed yearly in the United States [3,7]. As our total patient population bearing an IVC filter has grown, so have the known complications of IVC filters necessitating and complicating their retrieval.

IVC filters are designed with a retrievable option that allows them to be removed when no longer clinically indicated or when afflicted with a filter complication. Major filter complications include filter tilt, filter fracture, filter embolization and movement, IVC occlusion, and IVC penetration [1]. In such cases, there is a clear lack of consensus regarding management. Decisions are often made on a case-by-case basis with input from both vascular surgeons and interventional radiologists. The retrieval techniques range from simple to advanced and are fraught with issues influenced by the duration of the time that the patient has had the filter in situ, the presence of an embedded hook, and the tilt of the filter [8]. Retrieval procedures can cause significant IVC injury, fragmentation and migration of filter components into the heart or pulmonary vasculature, and filter strut penetration into the bowel, aorta, or kidneys [3]. Under these circumstances, a case that once appeared simple will escalate in terms of anesthetic and surgical management.

The role of the anesthesiologist in non-operating room locations has been expanding as procedures and patients become more complex with greater intraoperative management needs. However, most of the literature regarding procedural advancements and periprocedural requirements has been written by radiologists, with minimal consideration toward the complexities of case preparation for anesthesiologists. Here, we presented a case that highlights the anesthetic concerns of conducting a complex procedure on a sick patient while working in a non-operating room location. Our case is especially pertinent as the IR suite has already been shown to have a higher complication rate for patients receiving anesthesia than any other non-operating room anesthetizing location [9]. Contributing factors to consider include an unfamiliar work area with more limited resources, the absence of anesthesia technicians, and unfamiliar IR surgical protocols.

The Society of Interventional Radiology released its first-ever staffing guideline for the IR suite in 2016 [10]. As the role of the anesthesiologist continues to expand in IR cases, there remains a paucity of written guidelines to support when anesthesia is needed and under what context. As such, the available resources for anesthesiologists to properly staff complex cases of this type remain limited and require further consideration and development. We posit that this case illustrates the importance of in-depth planning, evaluation, and utilization of resources for anesthesiologists in a non-operating room setting.

## Conclusions

This case highlights three pertinent learning points. First, IVC filters can be quickly and efficiently retrieved through a variety of safe endovascular techniques. However, while straightforward, retrieval can still be high risk as associated complications include IVC penetration, IVC thrombosis, filter thrombosis, filter embolization and movement, and filter fracture. Second, the role of anesthesiologists in non-operating room cases is expanding as procedures and patients become more complex. Non-operating room locations are associated with higher patient complication rates and more limited resources for the anesthesiologist. Finally, complicated patients presenting for so-called “benign” surgical procedures in non-operating room locations beget the need for an extra layer of in-depth pre-operative planning and preparation with regards to resources.
